# Energy in the workplace: job demands, job resources, and employees’ inner resources as pathways to organizational outcomes

**DOI:** 10.3389/fpsyg.2024.1413901

**Published:** 2024-11-06

**Authors:** Cody R. DeHaan, Emma L. Bradshaw, Sandra Diaz-Castillo, Todd C. Trautman, C. Scott Rigby, Richard M. Ryan

**Affiliations:** ^1^Immersyve, Inc., Dallas, TX, United States; ^2^Institute for Positive Psychology and Education, Australian Catholic University, North Sydney, NSW, Australia; ^3^Evernorth Research Institute, St. Louis, MO, United States; ^4^Department of Education, Ewha Womans University, Seoul, Republic of Korea

**Keywords:** wellbeing, need support, self-determination theory, job demands-resources, Work

## Abstract

In this study, we expanded upon the job demands–resources model to assess the role of employees’ vitality as an inner resource for their work engagement and job commitment. To assess vitality and related job resources, we developed an index of vitality outside of work and adapted measures of manager autonomy support and organizational support. For job demands, we measured work stress and predicted that each of these four variables would contribute independently to work-related outcomes. Then, in a preregistered study, we collected these measures from a sample of 5,280 American workers (primarily ages 18–34, 54% female). Results from multivariate regression analyses largely confirmed our hypotheses, showing that positive work-related outcomes, such as enthusiasm, enjoyment, and job satisfaction, were positively predicted by manager autonomy supports, organizational support, and individuals’ vitality, and negatively predicted by work stress. The reverse pattern was largely observed for the negative outcome of turnover intention. Exploratory analyses also suggested that individual vitality may buffer the negative effects of stress and low manager and organizational support. The results highlight the potential role of employee vitality outside of work and managerial support in bolstering work engagement and reducing turnover intentions, offering a basis for organizational strategies aimed at improving work culture and retaining talent.

## Introduction

The energy employees have for engaging positively in their work is a growing area of study (Bakker and Albrecht, 2018). In particular, the *job demands–resources* (JD-R; Bakker and Demerouti, 2014) model has supplied a general framework for researching and understanding engagement and burnout in the workplace. The model specifies that certain job characteristics (i.e., “demands”) undermine staff engagement and retention (e.g., pressure and stress), while other characteristics (i.e., “resources,” like support and feedback) enhance work engagement and job commitment. Applying the JD-R model from the perspective of *self-determination theory* (SDT; Ryan and Deci, 2017) brings a focus to a specific set of contextual resources, including manager autonomy support and organizational support, as well as particular demands, like workplace pressure and stress. Also relevant to workplace thriving, however, may be the degree of intrapsychic energy from *non-work* sources that one brings to the workplace. Individual differences in people’s general vitality, confidence, and agency may present an *inner resource* pathway to optimal work outcomes. We tested these four resources and demands in relation to indicators of work engagement and job commitment to show each other’s unique contributions in order to further inform the multivariate nature of complex systems like the workplace.

### Job demand and resources

The JD-R model attempts to explain both the wellbeing and ill-health of employees via the dual pathways of job resources and job demands (Bakker and Demerouti, 2007; Xanthopoulou et al., 2007). Job resources are defined as the physical, psychological, social, or organizational aspects of the work context that (1) can reduce the depleting impact of job demands, (2) help employees achieve work goals, and/or (3) stimulate personal growth, learning, and development (Schaufeli and Bakker, 2004). Job resources are thus also expected to relate positively to wellbeing. The JD-R model specifically argues that job resources (e.g., managerial supports or opportunities for career development) are drivers of work engagement (Bakker and Demerouti, 2007, 2014) and varied positive organizational outcomes and key performance indicators (KPIs; Bakker et al., 2011). Indeed, evidence suggests that job resources such as job control, participation in decision-making, and task variety, have a positive impact on work engagement (e.g., Korunka et al., 2009; Kühnel et al., 2012) and, oppositely, a negative effect on burnout (e.g., Bakker et al., 2003; Schaufeli and Bakker, 2004).

Job demands refer to aspects of a job that require sustained physical and/or psychological effort and are therefore associated with physiological and/or psychological costs (Bakker and Demerouti, 2017). Demands are often represented in terms of workload, time pressures, and attentional demands that can be energy-depleting. In contrast to the positive effects of job resources, excessive job demands can lead to physical and psychological impairment and lower quality work motivation (Van Yperen et al., 2016).

### Job demands–resources and self-determination theory

SDT (Ryan and Deci, 2000, 2017) has identified three basic psychological needs that, when satisfied, enable human wellbeing and flourishing. Specifically, SDT argues that when employees experience autonomy (i.e., agency and volition), competence (i.e., ability and capacity), and relatedness (i.e., closeness with others) in their work, they perform at their best. Job resources that enhance these basic needs can help buffer or protect employees from depletion and burnout (e.g., Alarcon, 2011; Bakker et al., 2005) and enhance work engagement (e.g., Schaufeli et al., 2009), in part, because having access to such resources allows employees to satisfy their needs and increases their willingness to dedicate efforts and abilities to the work task (Bakker and Demerouti, 2007). In contrast, excessive job demands can lead to the frustration of basic psychological needs, as employees can feel controlled or less competent in such circumstances, potentially diminishing job satisfaction, enthusiasm, and engagement.

Exploring this interface between SDT and JD-R, Trépanier et al. (2015) found that job demands predicted high distress and psychosomatic complaints, low work engagement, and lower performance among employees. These outcomes were, as predicted, mediated by SDT’s basic need frustrations and by employees’ controlled (i.e., non-autonomous) motivation. In contrast, job resources positively predicted basic psychological need satisfaction and fostered more autonomous and less controlled employee motivation and functioning. The JD-R model also specifies that demands and resources can have *joint effects* on outcomes, such that the costs associated with some work demands can be balanced via the provision of various job-specific resources. SDT-based research has supported this claim from the JD-R model, showing that employees’ basic psychological need satisfaction can buffer the effects of both job demands and low resources on employee wellbeing (see Fernet et al., 2012a; Fernet et al., 2012b; Trépanier et al., 2013).

#### Manager autonomy support

SDT has a long history of motivational research in organizations, showing that basic psychological need satisfaction in one’s work climate enhances autonomous motivation (Baard et al., 2004; Mageau and Vallerand, 2003; Vansteenkiste et al., 2004). Among the main facilitators of such need satisfaction is *manager autonomy support*. In work settings, the interpersonal context is considered autonomy-supportive when managers provide a meaningful rationale for doing the tasks, emphasize choice rather than control, and acknowledge employees’ feelings and perspectives (Deci et al., 1989; Ryan and Deci, 2017). These behaviors are thought to foster an environment in which people are more likely to perceive their work as meaningful and personally relevant, making the workplace more worth investing in over the longer term. Indeed, past research has shown that autonomy-supportive managers foster greater autonomous motivation in their employees, which, in turn, predicts more positive work outcomes (e.g., Deci et al., 2001; Gagné et al., 2000). For instance, Hardré and Reeve (2009) showed, through an intervention-based experimental design, that when managers displayed an autonomy-supportive managerial style, employees were more autonomously motivated and engaged in work more than employees supervised by control-group managers. In a study of public sector employees, Kuvaas (2009) found that autonomy support positively predicted autonomous motivation, which was, in turn, related to better work performance. Recent meta-analyses have further supported these patterns of association (see Slemp et al., 2018; Van den Broeck et al., 2021).

#### Organizational support

A second source of support, a more distal one, comes from one’s general relationship with their employer or organization. *Perceived organizational support* is the degree to which employees believe that their organization values their contributions and cares about their wellbeing (Eisenberger et al., 1986; Eder and Eisenberger, 2008). Though the study of perceived organizational support has received considerable attention in the literature (see Rhoades and Eisenberger, 2002), fewer studies have looked at the role of perceived organizational support in the prediction of workers’ motivation, according to SDT. However, using samples from French industries, Gillet et al. (2013) found that perceived organizational support was associated with both more autonomous and more controlled work motivation, whereas supervisor autonomy support predicted only autonomous work motivation, which was, in turn, associated with lower turnover intention.

#### Workplace pressure and stress

In contrast to resources and supports that bolster experiences of need satisfaction and thus enhance work motivation and outcomes, the demands of work pressure and stress can detract from need satisfaction, and perhaps even actively frustrate those needs, leading to more detrimental outcomes. For example, Desrumaux et al. (2015) demonstrated a negative link between job demands and relatedness satisfaction, though they did not find a significant link for autonomy or competence. Job demands, conceptualized as task changes and ambiguity, were demonstrated to relate negatively to psychological need satisfaction in one study and positively to need satisfaction in another (Gillet et al., 2015).

#### Inner resources in the workplace: vitality, confidence, and agency outside of work

Although SDT research often highlights the environmental factors that impact motivation and psychological functioning, within SDT, it has also long been argued that individual differences influence how people perceive or react to their environment (Deci and Ryan, 1985). Alongside environmental factors, differences in motivational orientation, goals, resilience, and energy might well influence how employees adapt to job stressors and respond to the resultant strains and affordances of work life (see Frederick and Ryan, 2023; Ryan and Frederick, 1997). In the present research, we extend previous research combining the JD-R model and SDT, by assessing the individual differences in vitality, confidence, and agency that employees may possess outside of the workplace. We refer to this as an *inner resource* pathway to optimal organizational outcomes.

We define inner resources in the workplace context as attributes that individuals bring with them into the workplace, impacting their ability to handle various job demands. The index used here encompasses feelings of energy available to the self and feelings of autonomy and competence across various spheres of life, including social, emotional, intellectual, physical, environmental, occupational, and financial domains. In covering a wide variety of life domains, we aim to be comprehensive in capturing an individual’s overall available energy. We expected that these cross-domain perceptions of energy and agency would represent a robust set of inner resources concerning enthusiasm and engagement outside of work that, in turn, might influence job engagement beyond what organizational and managerial supports might afford.

We distinguish inner resources from a variety of outcomes, such as job satisfaction and enjoyment, as theoretical subjective experiences across a variety of life domains that would precede and potentially influence outcomes at work. In other words, we expect that work outcomes are specific to the workplace context and arise from the interplay of inner resources and the workplace environment. By focusing on these inner resources, we aim to demonstrate their ability to bolster workplace outcomes beyond the effect of day-to-day workplace factors.

The present research team was commissioned by Evernorth, with the aim of understanding the relation of job demands, job resources, and inner resources to people’s health and wellness. To this end, we employed the short form version of the Evernorth Vitality Index (EVI), referred to as the EVI-S, which assesses the domains described above. Details of the development of this scale are described in the Online Supplementary Materials S1, as the development of this scale is not the primary focus of this study. Before applying the new assessment in a formal and preregistered study, we first conducted an exploratory study to assess its strengths and characteristics.

### The current research

**Figure 1:**
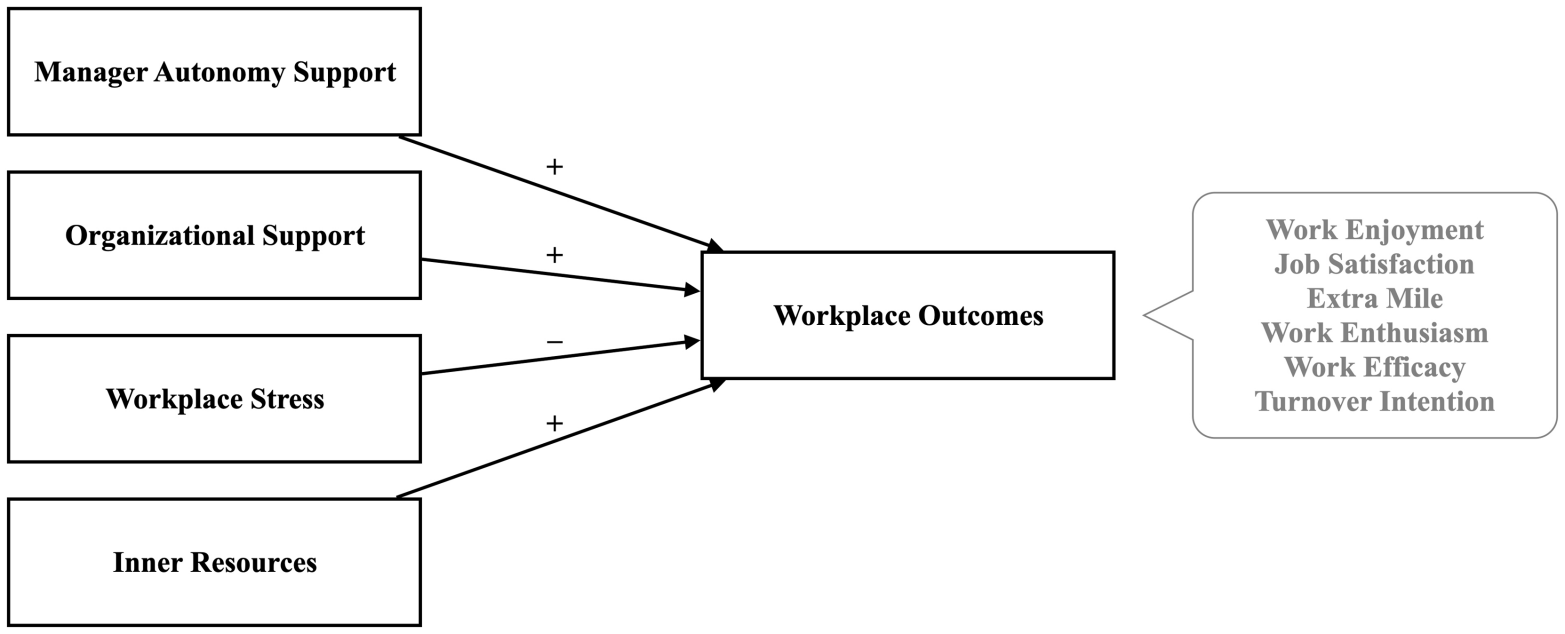
An overview of regression models in preliminary and preregistered studies.

Across two studies—an exploratory study and a preregistered study—we built toward our aim to test the combined JD-R and SDT model in which job and inner resources (as operationalized by manager autonomy support, organizational support, and vitality) alongside demands (operationalized as workplace pressure and stress) are tested simultaneously in relation to work engagement and job commitment. We conducted two studies as an initial foray into these relations. In the first, the exploratory study, we developed the composites and tested the models. In the second, the preregistered study, we tested the full preregistered models. Our hypotheses for the second, preregistered study, as outlined in Figure 1, were as follows:

*Hypothesis 1: Manager autonomy support will relate positively to work enjoyment, job satisfaction, extra mile, work enthusiasm, and work efficacy, and negatively to turnover intention, even when controlling for the other demands and resources in the model*.

*Hypothesis 2: Organizational support will positively relate to work enjoyment, job satisfaction, extra mile, work enthusiasm, and work efficacy, and negatively to turnover intention, even when controlling for the other demands and resources in the model*.

*Hypothesis 3: Workplace pressure and stress will negatively relate to work enjoyment, job satisfaction, extra mile, work enthusiasm, and work efficacy, and positively to turnover intention, even when controlling for the other demands and resources in the model*.

*Hypothesis 4: Inner resources, as measured by the EVI-S, will relate positively to work enjoyment, job satisfaction, extra mile, work enthusiasm, and work efficacy, and negatively to turnover intention, even when controlling for the other demands and resources in the model*.

## The exploratory study

### Method

#### Participants and procedure

The data for this preliminary study were collected by Morning Consult using a nationally representative sample in which 10,001 complete responses were collected, with the expectation that this sample would be a relatively diverse and comprehensive demographic broadly representative of the backgrounds and working conditions that would generalize to other developed economies (Table 1). In this sample, 57.5% (*n* = 5,755) were employed, and all analyses on the sample were limited to this subgroup. Of these, the 45–64 age group was the largest (37%), and most held a high school degree or greater. In addition, a wide variety of income brackets were represented, with most participants reporting a household income of $50,000–$74,999 (16%). Participants self-reported their gender (male, female, or prefer not to say), with responses summarized in Table 1.

**Table 1:** Demographics for the exploratory and preregistered studies.

	Exploratory study	Preregistered study
Responses	5,755	5,280
Age		
18–34	1,859 (32%)	2,188 (41%)
35–44	1,403 (24%)	1,100 (21%)
45–64	2,107 (37%)	1,772 (34%)
65+	386 (7%)	220 (4%)
Gender		
Male	2,875 (50%)	2,418 (46%)
Female	2,848 (50%)	2,840 (54%)
Prefer not to say	—	22 (<1%)
Employed	5,755 (100%)	5,280 (100%)
Household income		
<$25,000	559 (10%)	673 (13%)
$25,000–$34,999	581 (10%)	692 (13%)
$35,000–$49,999	632 (11%)	827 (16%)
$50,000–$74,999	915 (16%)	1,037 (20%)
$75,000–$99,999	885 (15%)	758 (14%)
$100,000–$124,999	660 (11%)	418 (8%)
$125,000–$149,999	606 (11%)	361 (7%)
$150,000–$199,999	445 (8%)	278 (5%)
>$200,000	315 (5%)	144 (3%)
Prefer Not to Answer	157 (3%)	92 (2%)
Education		
No Schooling	19 (<1%)	6 (<1%)
8^th^ Grade or Less	29 (1%)	13 (<1%)
Some High School, Did Not Graduate	124 (2%)	127 (2%)
High School/GED	957 (17%)	1,186 (22%)
Trade School/Vocational School Graduate	231 (4%)	275 (5%)
Some College or University Study	869 (15%)	920 (17%)
Associate’s Degree	581 (10%)	638 (12%)
Bachelor’s Degree	1,664 (29%)	1,339 (25%)
Master’s Degree	1,060 (18%)	651 (12%)
Doctorate Degree	221 (4%)	125 (2%)

#### Materials

Our study materials were a subset of those used for a larger study, which were intended for separate studies. Those variables were not used here, so they are not discussed further. As this study included a broad range of constructs, this study employed mostly face-valid, short measures.

##### Manager autonomy support

Manager autonomy support is the experience of managers providing a meaningful rationale, acknowledging employees’ perspectives, and encouraging questions. This job resource was assessed using five items on a 7-point scale (1 = *strongly disagree* to 7 = *strongly agree*), including items such as “I feel understood by my manager” and “My manager encourages me to ask questions.” Reliabilities were good (Cronbach’s α = 0.93), with all item loadings 0.83–0.86.

##### Organizational support

Organizational support is the perception that an organization values employees’ contributions and cares about their growth and wellbeing. Perceived organizational support, another job resource, was assessed using five items. Three of these items, such as “I am ____ with my chances for advancement on the job” were assessed on a 4-point satisfaction scale (1 = *very satisfied* to 4 = *not at all satisfied*; reversed for the composite). The remaining two items, such as “I am kept informed about what is going on in the company,” were assessed on a 4-point agreement scale (1 = *strongly agree* to *4* = *strongly disagree*; also reversed for the composite). The composite showed good reliabilities (Cronbach’s α = 0.82), with all items loading 0.66–0.74.

##### Workplace pressure and stress

Workplace pressure and stress are the experience of unreasonable deadlines, stress related to the job, and other negative experiences at the workplace. The job demand of workplace pressure and stress was assessed using three items on a 5-point frequency scale (1 = *never* to 5 = *very often*), including items such as “I have too many unreasonable deadlines” and “How often do you find your work stressful?.” The composite showed good reliabilities (Cronbach’s α = 0.73), with all items loading on a single factor at 0.56–0.81.

##### Inner resources

Inner resources are those resources individuals bring with them into the workplace, including energy, autonomy, and competence across various spheres of life. The short form version of the Evernorth Vitality Index (the EVI-S), as discussed in Online Supplementary Materials S1, was assessed using a 7-point scale (1 = *not at all true* to 7 = *very true*). Participants were given the instructions “Please read each statement carefully and indicate the degree to which each statement is true for you in general,” and items include “I feel alive and vital,” “I have all the skills and tools necessary to live a healthy life,” and “I feel capable of managing my emotions.” The index demonstrated good reliability (Cronbach’s α = 0.89), with item loadings on a single factor at 0.53–0.73.

##### Work enjoyment

The work enjoyment outcome was measured with a single item, “I value and enjoy my work,” on a 7-point scale (1 = *not at all true* to 7 = *very true*). For discrete variables, single-item measures are being increasingly considered useful due to the low participant burden they pose and their high correlations with their multi-item versions (Matthews et al., 2022).

##### Job satisfaction

The job satisfaction outcome was assessed with a single item, “Overall, I am ____ with my job,” on a 4-point scale (1 = *very satisfied* to 4 = *not at all satisfied*, reversed for interpretation).

### Results

All analyses were conducted in R version 4.3.1 (R Core Team, 2019), using packages including dplyr (1.1.2), psych (2.3.6), scipub (1.2.2), QuantPsyc (1.6), and FactoMineR (2.8). Correlations were calculated for all variables, as shown in Table 2. The outcomes were each analyzed using multivariate regression, in which all predictors (i.e., manager autonomy support, organizational support, inner resources, and work stress) were entered simultaneously. In addition, Funder and Ozer (2019) reviewed the psychological literature and suggested the benchmarks of *r* = 0.05, 0.10, 0.20, and 0.30 as indicative of very small, small, medium, and large effect sizes, respectively. We use these benchmarks in our discussion.

**Table 2:** Exploratory study correlations, means, and standard deviations.

	1	2	3	4	5	6
1. Inner resources	-					
2. MAS	0.30***	-				
3. Workplace stress	−0.08***	−0.18***	-			
4. Org. support	0.34***	0.58***	−0.20***	-		
5. Work enjoyment	0.59***	0.43***	−0.14***	0.45***	-	
6. Job satisfaction	0.41***	0.49***	−0.24***	0.69***	0.48***	-
7. Work–life balance	0.43***	0.58***	−0.17***	0.48***	0.40***	0.43***
Mean	5.04	4.93	2.70	3.06	5.29	3.31
SD	1.12	1.08	0.68	0.51	1.53	0.74

*p < 0.05, **p < 0.01, ***p < 0.001. MAS, manager autonomy support; Org. support, organizational support; SD, standard deviation.

#### Intercorrelations

The correlations between the variables are displayed in Table 2. The pattern of correlations largely conformed to our expectations. Inner resources were broadly positively associated with outcomes, as were manager autonomy support and organizational support. Furthermore, workplace stress was negatively associated with inner resources, as well as job satisfaction and work–life balance.

#### Gender effects

While we did not hypothesize any differences in the main study variables by gender, there were differences in all variables (see Table 3). These effects indicated that males, relative to females, experienced greater manager need support, organizational support, and inner resources, as well as greater workplace stress. In addition, males reported greater work enjoyment and job satisfaction.

**Table 3:** Exploratory study variable differences by gender.

Variable	*F*	df	*p*	Mean (Males)	Mean (Females)
Inner resources	118.0	1	<0.001	5.32	5.02
MAS	52.7	1	<0.001	5.02	4.75
Org. support	119.0	1	<0.001	3.20	3.01
Workplace stress	6.90	1	<0.01	2.76	2.70
Work enjoyment	21.3	1	<0.001	5.39	5.21
Job satisfaction	80.6	1	<0.001	3.40	3.23

MAS, manager autonomy support; Org. support, organizational support.

#### Regressions

##### Work enjoyment

As shown in Table 4, all four predictors (three job resources and one job demand) were statistically significantly associated with work enjoyment, with manager autonomy support (*β* = 0.14, *p* < 0.001), organizational support (*β* = 0.16, *p* < 0.001), and inner resources (*β* = 0.45, *p* < 0.001) all positively predicting work enjoyment, and workplace stress (*β* = −0.03, *p* < 0.01) exhibiting a small negative relation to work enjoyment. The overall *R^2^* for the model, with all four variables predicting work enjoyment, was 0.40.

**Table 4:** Exploratory study regressions with standardized coefficients.

	Work enjoyment	Job satisfaction
Manager autonomy support	0.14***	0.09***
Organizational support	0.16***	0.57***
Workplace stress	−0.03**	−0.09***
inner resources	0.45***	0.09***
Overall *R*^2^	0.40	0.50

*p < 0.05, **p < 0.01, ***p < 0.001.

##### Job satisfaction

All predictors were statistically significant in their associations with job satisfaction, with manager autonomy support (*β* = 0.09, *p* < 0.001), organizational support (*β* = 0.57, *p* < 0.001), and inner resources (*β* = 0.09 *p* < 0.001) all positively predicting work enjoyment, and workplace stress (*β* = −0.09, *p* < 0.001) negatively predicting job satisfaction. The overall *R*^2^ for the model, with all four variables predicting job satisfaction, was 0.50.

### Discussion

Manager autonomy support, organizational support, workplace stress, and inner resources all proved robust in explaining variance in work enjoyment, job satisfaction, and workplace stress consistent with our expectations. This suggests that the chosen independent variables are important to study outcomes, encouraging us to preregister and test with a broader array of outcomes. In addition, without overinterpreting the relative magnitude of coefficients, inner resources appeared to be particularly important for work enjoyment, whereas organizational support appeared to be the strongest predictor of job satisfaction. We thus felt confident in designing and preregistering a study testing the contribution of vitality alongside other job demands and resources in predicting an array of work outcomes, including willingness to go above and beyond requirements, enthusiasm for work, and turnover intention. This will serve as an extension of the outcomes associated with these independent variables and lend further support to the patterns revealed in the exploratory study.

## The preregistered study

### Method

#### Participants and procedure

##### Participants

A total of 10,000 responses were collected. Of the total sample, 5,280 were employed; further analyses were conducted only using this subsample. Participants were collected in a similar manner to the preliminary study. Most participants were from the 18–34 age group (41%), and most participants had a high school degree or greater. Participants self-reported their gender (male, female, or prefer not to say), with results summarized in Table 1.

##### Recruitment

Study participants were recruited by Morning Consult on behalf of Cigna Group and its subsidiaries, including Evernorth Research Institute. For the online survey, 10,000 adults ages 18 and over from the continental United States., Alaska, and Hawaii were interviewed online in English or Spanish between May and June, 2022. Recruitment was conducted among registered Dynata and Marketing Survey panel members using stratified sampling. To be eligible for participation, respondents had to be a member residing in the United States, be 18 years of age or older, voluntarily consent to participation, and be fluent in either English or Spanish.

To ensure that the sample was representative of the United States’ population, quotas were established based on Census Data using a cross-section of age and gender, with employment quotas based on Bureau of Labor Statistics data. To adjust for bias in respondent characteristics and contribute to representativeness, the sample was calibrated to the US population based on the US Census American Community Survey benchmarks on the known composition of the US adult population’s distribution by region, race, ethnicity, age, and gender and employment status. The survey had a margin of error of ±1% for the full 10,000 respondent sample. To further ensure sample quality and eliminate human bias, respondent quality measures were applied during the interviews. Quality checks included bot checks, timing tests, and open-ended questions, and to avoid missing data or implausible values, responses were required to questions.

##### Consent

Consent was obtained from each respondent via a “double opt-in” process. The panel was composed of individuals who opted to participate in surveys in exchange for an incentive payment. Individuals were contacted via email to participate in this specific survey. Participants first accepted the terms and conditions of participation, including detailed information on what data are collected and shared with research partners and how respondent data may be used. Once the recruitment questionnaire is completed, panelists receive an email and are required to click on the link from the email to confirm they would like to participate in panel membership (constituting the second “opt-in”).

##### Compensation

Compensation was offered for each participant. The form of compensation offered varied across respondents, given that the study leveraged several different sourcing mechanisms, each with its own approach to compensation. Fair market value compensation is given to all survey respondents. Compensation may have taken the form of cash, gift cards, and rewards points, among others. As a quality improvement initiative, the study did not constitute human subjects research in accordance with the Office of Human Research Protections guidance on Health and Human Services regulations at 45 CFR 46.102(d). All activities were conducted in accordance with the Marketing Research and Intelligence Association, Marketing Research Association, and Council of American Survey Research Organizations standards for North America, European Society for Opinion and Market Research (ESOMAR) and in compliance with the International Chamber of Commerce Code of Conduct on Market, Opinion, and Social Research and Data Analytics. This study was preregistered with the Open Science Framework at https://osf.io/48vs9/?view_only=8455c51d242b445a931252df8b760de5.

#### Materials

Our study materials were again a subset of those used for a larger study and intended for separate and unrelated analyses.

##### Manager autonomy support

The same measure was used as in the exploratory study, with an additional item included (for a total of six items) on a 7-point scale (1 = *strongly disagree* to 7 = *strongly agree*). The additional item, “My manager conveys confidence in my ability to do well at my job,” balanced the item set used in the exploratory study such that there were a matched number of positively and negatively worded items. Reliabilities were good (Cronbach’s α = 0.92), with all item loadings 0.75–0.84.

##### Organizational support

Perceived organizational support was assessed using four items. One of these items, “I am ____ with my chances for advancement on the job,” was assessed on a 5-point satisfaction scale (1 = *not at all satisfied* to 5 = *very satisfied*). An additional item, “I am kept informed about what is going on in the company,” was assessed on a 5-point agreement scale (1 = *strongly disagree* to *5* = *strongly agree*; also reversed for the composite). An additional item, “I work in an environment that is supportive of my family and personal commitments,” was assessed on a 7-point agreement scale (1 = *strongly disagree* to 7 = *strongly agree*). The final item, “I receive appropriate recognition for good performance,” was assessed on a 5-point frequency scale (1 = *never* to 5 = *very often*). All items were rescaled on a 7-point scale before combining. The composite showed acceptable reliabilities (Cronbach’s α = 0.73), with all items loading 0.60–0.70.

##### Workplace pressure and stress

The workplace stress composite was assessed using four items on a 5-point frequency scale (1 = *never* to 5 = *very often*), including items such as “I have too many unreasonable deadlines” and “How often do you find your work stressful?.” The composite showed good reliabilities (Cronbach’s α = 0.73), with all items loading on a single factor at 0.42–0.73.

##### Inner Resources

Inner resources, as in the exploratory study, were assessed using the 10-item EVI-S on a 7-point scale (1 = *not at all true* to 7 = *very true*). The items used assessed general life vitality, as well as items assessing physical, social, intellectual, emotional, financial, and environmental agency. The index demonstrated good reliability (Cronbach’s α = 0.89) and item loadings on a single factor at 0.51–0.77.

##### Work enjoyment

Work enjoyment was measured with a single item, “I value and enjoy my work” on a 7-point scale (1 = *not at all true* to 7 = *very true*).

##### Job satisfaction

Job satisfaction was assessed with a single item, “Overall, I am ____ with my job” on a 5-point scale (1 = *not at all satisfied* to 5 = *very satisfied*).

##### Extra mile

Willingness to go the extra mile was assessed with a single item, “I am willing to work harder than I have to in order to help my workplace succeed” on a 5-point agreement scale (1 = *strongly agree* to 5 = *strongly disagree*, reversed for interpretation).

##### Work enthusiasm

Work enthusiasm was assessed using a single item, “I am enthusiastic about my job” on a 5-point scale (1 = *strongly agree* to 5 = *strongly disagree*, reversed for interpretation).

##### Work efficacy

Work efficacy was assessed using a single item, “How would you rate your ability to carry out your duties at work in the past month, using a scale from 1 to 7 where 7 means you were able to carry out your duties extremely well, and 1 means you were not able to carry out your work duties well at all?” Responses were on a 7-point scale (1 = *not well at all* to 7 = *extremely well*).

##### Turnover intention

Turnover intention was assessed using a single item, “Taking everything into consideration, how likely is it you will make a genuine effort to find a new job with another employer within the next year?” Responses were on a 7-point scale (1 = *very likely* to 5 = *very unlikely*).

#### Common method variance

As this study relied fully on self-report data, common method variance can be a concern. Common method variance is attributable to the method of measurement as opposed to the actual constructs of interest (Podsakoff et al., 2003). It can change the nature of relations among variables, leading to biased results. We employed Harman’s (1976) one-factor test to check for common method variance in the preregistered study, with an unrotated factor analysis indicating that the single-factor solution accounted for approximately 30% of the variance, below the recommended 50% threshold (Podsakoff and Organ, 1986). This provides reassurance that common method variance is not a critical issue here, although it is nonetheless a limitation of this work.

### Results

#### Intercorrelations

Correlations were calculated for all variables and outcomes, as shown in Table 5. The intercorrelations were as expected and in similar patterns to those described in the exploratory study. The outcomes were each analyzed using a multivariate regression in which all predictors (i.e., manager autonomy support, organizational support, inner resources, and work stress) were entered simultaneously. Results are summarized in Table 6.

**Table 5:** Preregistered study correlations, means, and standard deviations.

	1	2	2	4	5	6	7	8	9	10
1. Inner resources	-									
2. MAS	0.42***	-								
3. Org. Support	0.48***	0.67***	-							
4. workplace stress	−0.08***	−0.12***	−0.07***	-						
5. Work enjoyment	0.60***	0.41***	0.47***	−0.07***	-					
6. Job satisfaction	0.42***	0.48***	0.61***	−0.17***	0.51***	-				
7. Extra mile	0.30***	0.33***	0.37***	0	0.33***	0.34***	-			
8. Work enthusiasm	0.40***	0.41***	0.47***	−0.13***	0.50***	0.54***	0.56***	-		
9. Work efficacy	0.44***	0.34***	0.38***	−0.06***	0.35***	0.34***	0.29***	0.35***	-	
10. Turnover intention	−0.03	−0.13***	−0.18***	0.16***	−0.12***	−0.26***	−0.06***	−0.16***	−0.14***	-
Mean	5.04	4.8	4.57	3.31	5.11	2.31	2.20	2.33	5.45	3.06
SD	1.08	1.38	1.24	0.81	1.56	1.09	1.05	1.12	1.40	1.40

*p < 0.05, **p < 0.01, ***p < 0.001. MAS, manager autonomy support; Org. support, organizational support.

**Table 6:** Preregistered study regressions.

	Work enjoyment	Job satisfaction	Extra mile	Work enthusiasm	Work efficacy	Turnover intention
Manager autonomy support	0.09***	0.08***	0.13***	0.13***	0.09***	−0.01, ns
Organizational support	0.18***	0.49***	0.22***	0.27***	0.17***	−0.20***
Workplace stress	−0.01, ns	−0.12***	0.04**	−0.11***	−0.02, ns	0.15***
Inner resources	0.47***	0.14***	0.14***	0.22***	0.32***	0.09***
Model *R*^2^	0.40	0.41	0.16	0.28	0.23	0.06

*p < 0.05, **p < 0.01, ***p < 0.001.

#### Age effects

There were age group differences on several study variables, as shown in Table 7. In general, the youngest age group (18–34) had the lowest availability of inner resources, experienced the lowest organizational support, and broadly reported low work enjoyment, enthusiasm, and efficacy. They were also the most likely to look for another job. In contrast, those 65+ had the greatest inner resources, experienced the highest organizational support, and broadly enjoyed their work. Notably, the 45–64 group reported similarly low levels of organizational support and work enjoyment to the 18–34 group.

**Table 7:** Preregistered study exploratory differences by age.

Variable	*F*	df	*p*	18–34	35–44	45–64	65+
Inner resources	7.51	3	<0.001	4.99	5.07	5.05	5.33
Manager need support	1.35	3	0.255	4.78	4.87	4.78	4.80
Organizational support	3.73	3	0.011	4.53	4.65	4.55	4.71
Workplace stress	8.04	3	<0.001	3.31	3.33	3.33	3.05
Work enjoyment	8.92	3	<0.001	5.05	5.22	5.07	5.54
Job satisfaction	9.25	3	<0.001	3.70	3.70	3.63	4.04
Extra mile	8.65	3	<0.001	3.71	3.84	3.87	3.87
Work enthusiasm	6.75	3	<0.001	3.62	3.67	3.70	3.96
Work efficacy	75.82	3	<0.001	5.14	5.48	5.70	6.15
Turnover intention	126.40	3	<0.001	3.25	3.09	2.59	1.90

#### Gender effects

There were also gender differences in several study variables, as shown in Table 8. These effects largely replicated those reported in the preliminary study. Males, relative to females, reported greater inner resources; the manager needs support and organizational support. However, in contrast to the preliminary study, males reported lower work stress. Males also reported higher on all outcomes, except for work efficacy, for which there were no gender differences.

**Table 8:** Preregistered study exploratory differences by gender (males and females only).

Variable	F	df	*p*	Mean (Males)	Mean (Females)
Inner resources	37.2	1	<0.001	5.14	4.96
Manager need support	12.7	1	<0.001	4.87	4.74
Org. support	23.9	1	<0.001	4.66	4.49
Workplace stress	5.15	1	0.023	3.28	3.33
Work enjoyment	6.40	1	0.012	5.17	5.06
Job satisfaction	25.4	1	<0.001	3.78	3.62
Extra mile	7.86	1	<0.01	3.85	3.76
Work enthusiasm	6.89	1	<0.01	3.72	3.64
Work efficacy	0.32	1	0.57	5.45	5.43
Turnover intention	18.0	1	<0.001	3.03	2.86

Org. support, organizational support.

#### Preregistered analyses

##### Work Enjoyment

Manager autonomy support (*β* = 0.09, *p* < 0.001), organizational support (*β* = 0.18, *p* < 0.001), and inner resources (*β* = 0.47, *p* < 0.001) were all statistically significant, positive predictors of work enjoyment. Workplace pressure and stress were not related to work enjoyment (*β* = −0.01, *p* = 0.364). The overall *R^2^* for the model, with all four variables predicting work enjoyment, was 0.40.

##### Job satisfaction

Manager autonomy support (*β* = 0.08, *p* < 0.001), organizational support (*β* = 0.49, *p* < 0.001), and inner resources (*β* = 0.14 *p* < 0.001) all statistically significantly, positively predicted job satisfaction, and workplace pressure and stress (*β* = −0.12, *p* < 0.001) negatively predicted job satisfaction. The overall *R^2^* for the model, with all four variables predicting job satisfaction, was 0.41.

##### Extra mile

Manager autonomy support (*β* = 0.13, *p* < 0.001), organizational support (*β* = 0.22, *p* < 0.001), and inner resources (*β* = 0.14 *p* < 0.001) all statistically significantly, positively predicted going the extra mile. Interestingly, but unexpectedly, workplace pressure and stress (*β* = 0.04, *p* < 0.01) were also positively related to the extra mile index. The overall *R^2^* for the model with all four variables predicting an extra mile was 0.16.

##### Work enthusiasm

Manager autonomy support (*β* = 0.13, *p* < 0.001), organizational support (*β* = 0.27, *p* < 0.001), and inner resources (*β* = 0.22 *p* < 0.001) all statistically significantly, positively predicted enthusiasm, and workplace pressure and stress (*β* = −0.11, *p* < 0.001) negatively related to this variable. The overall *R^2^* for the model, with all four variables predicting work enthusiasm, was 0.28.

##### Work efficacy

Manager autonomy support (*β* = 0.09, *p* < 0.001), organizational support (*β* = 0.17, *p* < 0.001), and inner resources (*β* = 0.32 *p* < 0.001) all statistically significantly, positively predicted work efficacy. Workplace stress was not related to work efficacy (*β* = −0.02, *p* = 0.210). The overall *R^2^* for the model, with all four composites predicting work efficacy, was 0.23.

##### Turnover intention

Not all predictors were statistically significant, with manager autonomy support unrelated to turnover intention (*β* = −0.01, *p =* 0.498). Organizational support (*β* = −0.20, *p* < 0.001) was negatively related to turnover intention, whereas workplace stress was positively related (*β* = 0.15, *p* < 0.001). Interestingly, inner resources also predicted turnover intention (*β* = 0.09, *p* < 0.001), but positively. The overall *R*^2^ for the model with all four composites predicting turnover intention was 0.06, making it the most weakly predicted outcome.

### Exploratory analyses

In the interest of fully elucidating the dynamics present in these data, we conducted a small set of exploratory analyses that were not hypothesized ahead of time and thus not preregistered. All analyses concerned moderation effects: first, whether inner resources moderate the impact of job resources or demands in relation to workplace outcomes; second, whether factors such as employee age, industry, or gender moderated the main effects observed (Table 9).

**Table 9:** Preregistered study exploratory differences by industry group.

Variable	Inner resources	Manager autonomy Support	Organizational support	Workplace stress	Work enjoyment	Job satisfaction	Extra mile	Work enthusiasm	Turnover intention
*F*	11.35	11.32	21.90	10.03	5.53	10.15	5.21	6.13	18.86
df	5	5	5	5	5	5	5	5	5
*p*	<0.001	<0.001	<0.001	<0.001	<0.001	<0.001	<0.001	<0.001	<0.001
Health and biotechnology	5.04	4.83	4.58	3.42	5.22	3.74	3.79	3.73	2.81
Industrial and construction	5.08	4.76	4.57	3.23	5.17	3.73	3.86	3.67	2.83
Other	4.94	4.71	4.43	3.22	4.99	3.62	3.76	3.57	2.85
Professional and business services	5.24	5.08	4.90	3.35	5.27	3.87	3.94	3.80	3.24
Public and infrastructure	5.03	4.73	4.55	3.39	5.08	3.68	3.73	3.70	2.72
Service and hospitality	4.92	4.68	4.37	3.25	4.99	3.55	3.75	3.58	3.09

#### Inner resources as a moderator

One question that arose after conducting the hypothesized analyses was whether inner resources, as measured by the EVI-S, could function as a moderator for any of the effects of work demands or resources on outcomes. Accordingly, using regression analysis, we examined each of the above-reported effects with an additional interaction effect between each of the predictor variables and inner resources on the outcome variable. Each model thus included four main effects and three interaction terms. For purposes of this exploratory analysis, all outcomes are reported in Table 10, and plots of these interactions are included in the Online Supplementary Materials S3. The results for going the extra mile and work efficacy were not statistically significant and are not outlined below.

**Table 10:** Preregistered study exploratory regressions including interactions.

	Work enjoyment	Job satisfaction	Extra mile	Work enthusiasm	Work efficacy	Turnover intention
MAS	0.11	−0.01	0.19*	0.00	0.18*	0.06
Org. support	0.36***	0.53***	0.23**	0.42***	0.10	−0.56***
WP stress	0.03	−0.33***	0.08	−0.18***	0.02	−0.29***
Inner Resources	0.65***	−0.07	0.23**	0.13	0.36***	−0.50***
Inner resources * MAS	0.00	0.01	−0.01	0.02	−0.01	−0.01
Inner resources * Org. Support	−0.04**	−0.01	0.00	−0.03*	0.01	0.07***
Inner resources * WP stress	−0.01	0.06***	−0.01	0.03*	−0.01	0.13***
Model *R*^2^	0.40	0.42	0.16	0.28	0.23	0.07

Org. support, organizational support; MAS, manager autonomy support; WP stress, workplace stress.

##### Work enjoyment

For work enjoyment, only the inner resources by organizational support term was statistically significant. The interaction effect was probed using simple slopes analysis (Aiken and West, 1991). The slope of organizational support was statistically significantly different from zero at 1 SD above the mean of inner resources (0.18, *p* < 0.001), as well as −1SD, the mean of inner resources (0.28, *p* < 0.001). This pattern suggests that organizational support has a stronger effect on work enjoyment when inner resources are below average relative to when inner resources are above average.

##### Job satisfaction

For job satisfaction, only the inner resources by workplace pressure and stress term were statistically significant. Simple slopes revealed that the slope of workplace stress was different at +1SD above the mean of inner resources (−0.10, *p* < 0.001), as well as −1SD, the mean of inner resources (−0.22, *p* < 0.001). This pattern supports a buffering effect of inner resources, suggesting that workplace stress has a weaker negative effect on job satisfaction when inner resources are high.

##### Work enthusiasm

For work enthusiasm, two interactions were statistically significant. The first, inner resources by workplace pressure and stress, indicated that the slope of workplace stress was different from zero at +1SD, the mean of inner resources (−0.07, *p* < 0.001), as well as −1SD, the mean of inner resources (−0.14, *p* < 0.001). This pattern suggests that workplace stress has a more negative effect on work enthusiasm when inner resources are below average relative to when inner resources are above average. The second statistically significant interaction was inner resources through organizational support. The simple slopes suggested that organizational support was different from zero at +1SD, the mean of inner resources (0.22, *p* < 0.001), as well as −1SD, the mean of inner resources (0.27, *p* < 0.001), suggesting that organizational support had a slightly stronger impact on work enthusiasm when inner resources are below average relative to above average.

##### Turnover intention

For turnover intentions, two interactions were statistically significant. The first was inner resources by organizational support. The simple slopes suggested that the slope of organizational support was different from zero at +1SD, the mean of inner resources (−0.15, *p* < 0.001), as well as −1SD, the mean of inner resources (−0.32, *p* < 0.001). This pattern suggests that a lack of organizational support has a stronger impact on turnover intention when individuals have low inner resources relative to high inner resources. The second significant interaction was inner resources, which interacted with workplace pressure and stress. The simple slopes suggested that the effect of workplace stress was different from zero at +1SD, the mean of inner resources (0.41, *p* < 0.001), as well as −1SD, the mean of inner resources (0.09, *p* < 0.05). This pattern indicates that workplace stress has a higher impact on turnover intention when inner resources are high.

## Discussion

Workplace wellbeing, energy, and effort are multidetermined outcomes. In this study, our focus was on assessing how job demands and resources impact these outcomes across a range of occupations and industries, findings that support the generalizability of the JD-R model. Furthermore, we aimed to add to this picture the role that individual differences in vitality and agency (i.e., inner resources) might play in relation to these outcomes. Results of the preregistered components of these studies generally supported the four hypotheses concerning the positive links between manager autonomy, organizational support, and inner resources, with optimal organizational outcomes, and negative links with workplace pressure. We showed that the inner resource of vitality from non-work sources was associated with more work engagement and job commitment, even accounting for the other demands and resources in the models. These findings support the notion that the non-work inner resources that individuals bring to the workplace may buffer the effects of job demands or stressors. The workplace implications of these results are that employers may benefit from considering not only job resources and demands but also the inner resources that employees bring to work.

### The effects of job demands and resources

Our evidence lends support to the proposition that manager autonomy support is positively associated with beneficial work-related outcomes as work enjoyment, job satisfaction, and the willingness to go beyond the call of duty. However, it may not relate to employees’ intentions to leave their positions. Meanwhile, organizational support positively related to all beneficial outcomes and, crucially, negatively related to turnover intention. When employees perceive clear communication about their company’s trajectory and recognize opportunities for growth—key components of organizational support—they are more likely to be engaged with and satisfied at work. It also suggests that manager autonomy support alone may be insufficient to reduce turnover among staff if they do not also experience support at the level of the organization.

Although workplace pressure and stress are linked to a decline in job satisfaction and enthusiasm and an increase in turnover intention, these factors also appeared to, perhaps counterintuitively, relate to employees’ drive to exceed performance expectations. From an SDT standpoint, such an effect may be a function of introjected regulation, whereby pressure and stress result in people seeking approval from their leaders (Deci et al., 2017), which can be motivating in the short term but tends not to be related to performance outcomes (Zhang et al., 2016). Additionally, workplace pressure and stress did not significantly impact work enjoyment nor work efficacy when entered alongside manager supports and organizational supports. This was found despite significant zero-order correlations with these variables, suggesting that workplace pressure and stress may be less tightly linked to work enjoyment and efficacy than organizational and manager supports. It seems to suggest that supports need to be present at multiple levels of a workplace, and the right balance of optimal challenge needs to be struck to engage and retain satisfied staff. The complexity of these dynamics also suggests that employee wellness should not be an afterthought or limited to sporadic initiatives that are too peripheral to engage with meaningfully. Rather, wellbeing should underpin a workplace culture that addresses basic psychological needs, provides growth opportunities, and recognizes efforts, thereby weaving wellbeing into the fabric of daily work life.

### The effects of non-work resources: inner resources

Inner resources, as measured by the newly developed Evernorth Vitality Index, were shown to relate beneficially to workplace outcomes. Hypothesis 4 was partially supported, with inner resources relating to increased work enjoyment, job satisfaction, extra mile, work enthusiasm, and work efficacy. However, contrary to the hypotheses, inner resources were also positively related to turnover intention in the model. Possibly, feelings of vitality, agency, and capability in non-work domains make people more open and receptive to other workplace opportunities. In general, however, it seems that people’s inner resources across the physical, emotional, intellectual, social, environmental, and financial domains provided an indicator of employee wellness beyond work-related indicators. Yet, it was relevant to a variety of important workplace outcomes. Indeed, the inner resource pathway to positive employee outcomes may prove fruitful for human resource management because our exploratory analyses showed that inner resources might buffer against job demands and, therefore, could be a useful target for workplace interventions. This research suggests considering a broader spectrum of employee experiences—and providing supports for employees outside of the workplace—can bolster employee wellness and performance at work.

Previous studies have underscored the interconnectedness of work and non-work aspects of people’s lives and their dual influences on employee wellbeing and productivity (Beauregard et al., 2011; Sanhokwe, 2022; Sørensen et al., 2021). Our findings support those claims and suggest that a comprehensive approach to understanding employee wellbeing and work outcomes that encompasses both work- and non-work-related demands and resources may be most beneficial to organizations and the people they employ.

### Exploratory analyses

Exploratory analyses were added to this story. Inner resources not only had main effects on outcomes but also played a buffering role when job stress was high, or organizational and manager supports were low. In fact, the interaction results suggested that demands and resources make more of a difference for employees who are low in inner resources, having more impact on their job enthusiasm, job satisfaction, and work enjoyment. Such findings suggest that inner resources are a source of resilience for workers. However, these exploratory analyses also suggested that, when overly stressed, employees with high vitality are at risk of turnover, perhaps because they have the agency and energy to consider alternatives. Yet we note that, although these moderation effects generally appear reliable and the sample is large, they should be cautiously interpreted since they were not specified *a priori* in our registered hypotheses, and they need to be tested in longitudinal studies to determine the direction of causation.

Furthermore, industry data were collected and grouped (see Online Supplemental Materials S2). All study variables were compared to understand if industry categories differed on key study variables. All comparisons were statistically significant, with relatively consistent patterns of results across variables. Notably, the Service and Hospitality category reported low job resources and low positive outcomes. In contrast, Professional and Business Services reported greater inner resources, greater job resources, and higher positive outcomes. These findings offer practical implications for managers and organizations, by providing insights that encourage organizations to address non-work factors. Proactively understanding and addressing both work and non-work domains together can contribute to creating value for both individuals and businesses.

### Limitations

One of the limitations of this study is that we relied primarily on adapted and short-form versions of measures both to reduce participant burden and facilitate the future use of the measures in commercial settings, where brevity is important. The future study would ideally replicate these results and more fully embed them in the extant literature using validated measures where possible. In addition, considering multiple measurement timepoints can reduce recall biases, and continuing to ensure participant anonymity will reduce any social desirability demands. Furthermore, a wider variety of outcomes could be considered, including more objective measures of, for example, job performance and turnover. We do believe self-reporting will continue to be a core method of this work, given the importance of subjective experiences in these models.

The current study also does not establish the causality of effects in the model. While job resources (via manager autonomy support and organizational support), job demands (via workplace stress), and inner resources (EVI-S) were treated in the preregistered model as simultaneous predictors of outcomes, there remains the possibility of a deeper process model here. Future studies should build from these cross-sectional foundations and explore the causal pattern of results to explore if these relations hold over time, particularly if changes in inner resources, job resources, and job demands produce meaningful changes in outcomes at subsequent times. Additional objective outcomes could be considered as well, including any reliable and defensible measures of workplace outcomes or, across a large enough population, actual turnover rates.

The exploratory models with inner resources serving as a moderator of the effects the other variables have on outcomes spark interest in considering more nuanced models of these dynamics. In particular, future studies can explore the kinds of offerings and interventions workplaces can provide that bolster inner resources, leading to greater wellness and performance at work and—presumably—outside of it.

It is also noteworthy that our samples were limited to US workers, and generalizability to employees in other cultures is not established. In addition, we looked at a restricted number of variables under the JD-R categories of resources and demands. A more comprehensive assessment of these categories would undoubtedly account for more variance and supply comparative information on the relative strength of predictors in the workplace.

### Conclusion

In sum, we were able to demonstrate the important role of job climate in wellbeing and distress at work, as understood by the JD-R framework. Both the job resources of organization support and manager autonomy support were associated with better job outcomes, with job demands generally being a negative predictor. These results thus support the JD-R framework and highlight that the inner resources of the individual matter with regard to work outcomes as they turned out to be strongly correlated with optimal functioning at work. This study then contributes to both the JD-R theory and SDT. With respect to JD-R, we expand the scope of the broad category of resources to include individual differences. Although we highlighted vitality and agency here, other inner resources could be examined in future research within the JD-R framework, expanding opportunities for research on work-related wellbeing, job satisfaction, and performance. With respect to SDT, these results further establish the importance of vitality and agency as inner resources that both yield positive outcomes directly and may also help as a buffer against frustrating elements in one’s world.

## Data Availability

The dataset was privately sourced, and remains privately managed by Evernorth Research Institute. Requests to access the datasets should be directed to richard.ryan@acu.edu.au.
