# Supplementary material for: Energy in the workplace: job demands, job resources, and employees’ inner resources as pathways to organizational outcomes
Source: Front Psychol. 2024 Nov 6;15:1413901. doi: 10.3389/fpsyg.2024.1413901 (PMC11576300; doi:10.3389/fpsyg.2024.1413901)
Supplement: Supplementary file 1 [file Data_Sheet_1.docx]

# Supplemental Materials

## S1. Inner Resources Measure (EVI and EVI-S) Development

We developed an item pool with which we asked about people’s vitality and autonomy and competence within multiple spheres of life including emotional, intellectual, social, financial, spiritual, occupational, environmental, and physical areas. Together these items reflected both an experience of energy and of having the capacity to put it to use. Autonomy and competence items were inspired by extant measures of basic psychological need satisfaction (e.g., Chen et al., 2015), whereas items tapping feelings of energy available to the self were adapted from the Subjective Vitality Scale (SVS; Frederick & Ryan, 2023; Ryan & Frederick, 1997).

Beginning with a larger pool of 56 items tapping vitality, autonomy, and competence, in a preliminary sample we selected those with the best psychometric characteristics (appropriate variability, internal consistency etc.) The items were analyzed based on their a priori conceptual domains, as opposed to using methods to determine the number of components or factors analytically, due to the number of domains involved. For each domain a parallel analysis (Hayton et al., 2004) was conducted, which includes both a principal component analysis and a factor analysis. Ultimately, we created a 28-item score with 2 competence and 2 autonomy items for each of six domains (emotional, intellectual, social, financial, environmental, and physical areas of life), and 4 items tapping general energy for a total of 28 items. This 28-item full scale showed excellent internal consistency and preliminary construct validity in relation to other well-being and ill-being variables.

As expected from prior research, the autonomy, competence, and vitality items were strongly interrelated, and the total set of items showed high internal coherence (⍺ > .90). However, it was also clear that our spirituality items were the least coherent with each other and with the other subscales, and a subset of participants appeared not to endorse these items. We thus decided to create a summary score excluding the spirituality domain. Moreover, because we were interested in agency and vitality across all individuals, whether employed or not, we also did not include items from the occupational domain. This would allow the use of the index not only to workers but also to others such as students, unemployed persons, and retired individuals in the general population. Further, and important to the current studies, this design allowed us to better target in the inner resources a person might *bring* to the job, rather than vitality and agency experienced within, or derived from their work. That is, we wanted our index to reflect a person’s agency and energy in life generally, rather than in a work context per se.

Finally, we wanted to distill a useful index for applied work surveys, in which a 28-item questionnaire poses challenges. Typically, employees resist long surveys with redundant items. Thus, to create a practical short form, we subjected these 28 items to a *genetic algorithm* (GA; Sohail, 2023; Whitley, 1994) procedure. This GA procedure selected a shorter 10-item subset of the item pool that was maximally predictive of variance in the full item set, and is referred to as the EVI-S. Specifically, the GA produced a 10-item solution that correlated with the full scale at *r* = 0.97, suggesting they were nearly identical. The reduced scale also evidenced excellent internal consistency (Cronbach’s alpha = 0.89). A parallel analysis, focused on principal components analysis, found that the scale comprised a single principal component.

## S2. Industry Mapping

1. **Service and Hospitality Sector:**

- Hospitality/Restaurants (lodging, food services, travel, and tourism)
- Arts, entertainment, and recreation (writer, museum, event promotion, etc.)
- Real estate (sales, rental, leasing, etc.)
- Retail/consumer goods (e-commerce/big box stores)

1. **Industrial and Construction Sector:**

- Construction or trade (electrical, plumbing, etc.)
- Manufacturing, of any kind
- Utilities (electric power generation, transmission, distribution)

1. **Health and Biotechnology Sector:**

- Healthcare, hospitals, clinical settings/offices, labs
- Pharmaceuticals, biotechnology, life sciences

1. **Professional and Business Services Sector:**

- Financial services, banking, and insurance
- Marketing, advertising, or public relations
- Professional/business services (consulting, freelancing, legal, accounting, etc.)
- Technology/IT/telecommunications (software development, computer programming, etc.)

1. **Public and Infrastructure Sector:**

- Education (Primary/Secondary)
- Education (Vocational/Technical, Trade, College, and Post-Graduate)
- Government (local, state, federal, active-duty armed forces, etc.)
- Transportation and Warehousing (transit, airlines, couriers, messengers, etc.)

## S3. Interaction Plots for Exploratory Analyses in the Preregistered Study

All of these plots are generated using *interact_plot* from the *interactions* (version 1.2.0) package for R (version 4.4.0). The standard plot points of +/- 1 standard deviation from the mean are used in each.

### Work Enjoyment

Figure S3.1. Plot of the interaction of Organizational Supports and EVI predicting Work Enjoyment.


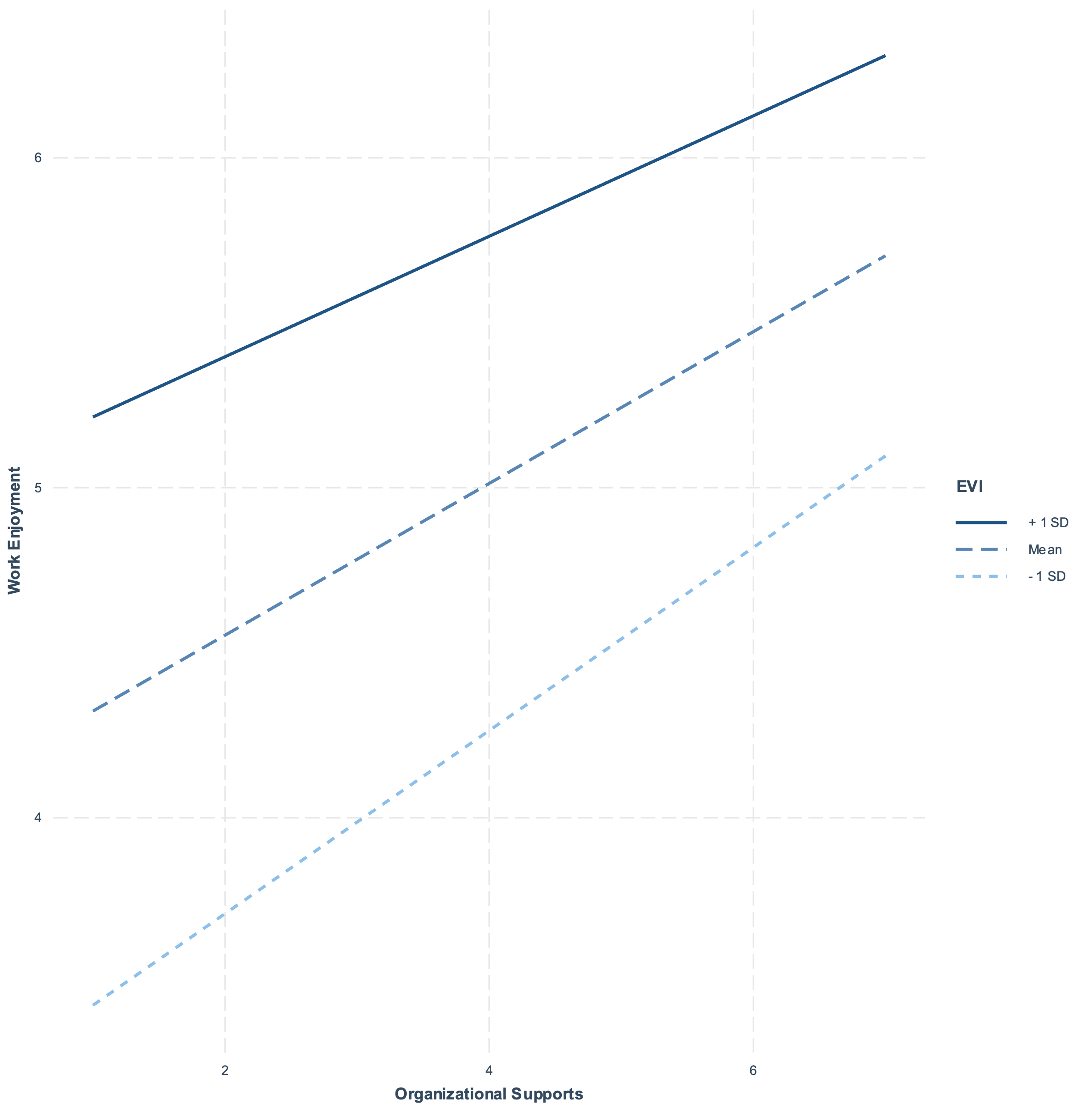


### Job Satisfaction

Figure S3.2. Plot of the interaction of Workplace Stress and EVI predicting Job Satisfaction.


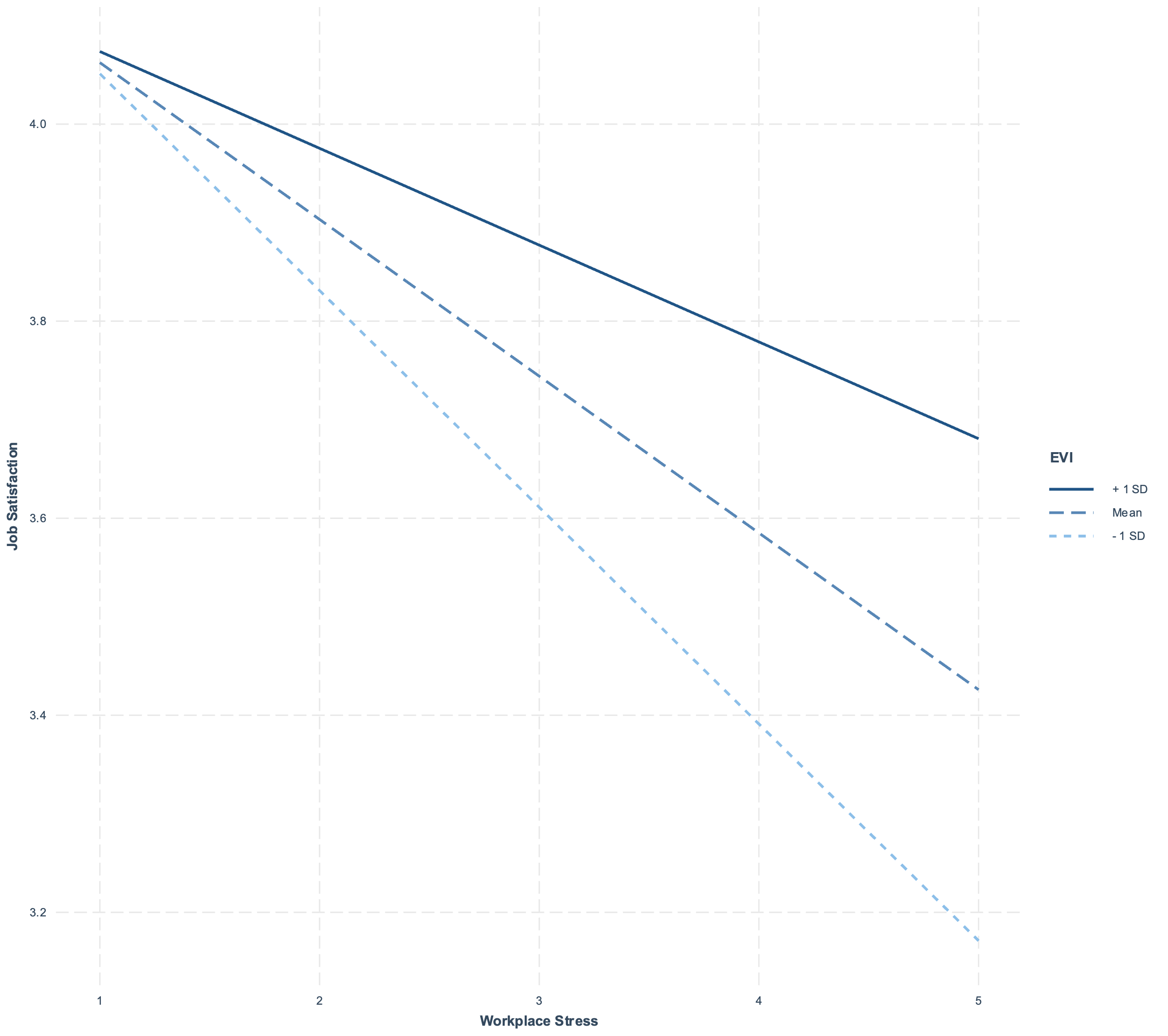


### Work Enthusiasm

Figure S3.3. Plot of the interaction of Workplace Stress and EVI predicting Work Exhaustion.


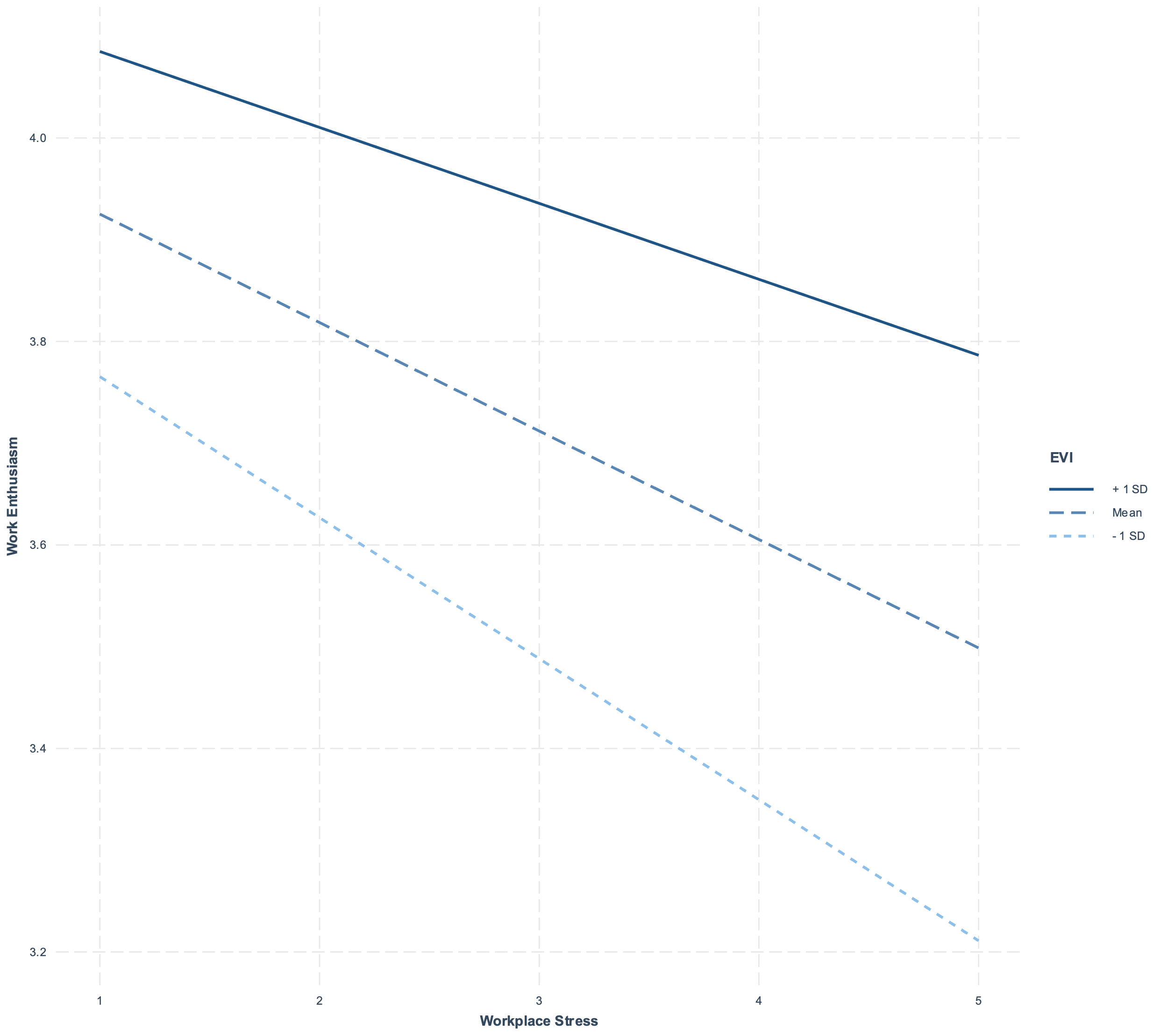


Figure S3.4. Plot of the interaction of Organizational Supports and EVI predicting Work Exhaustion.


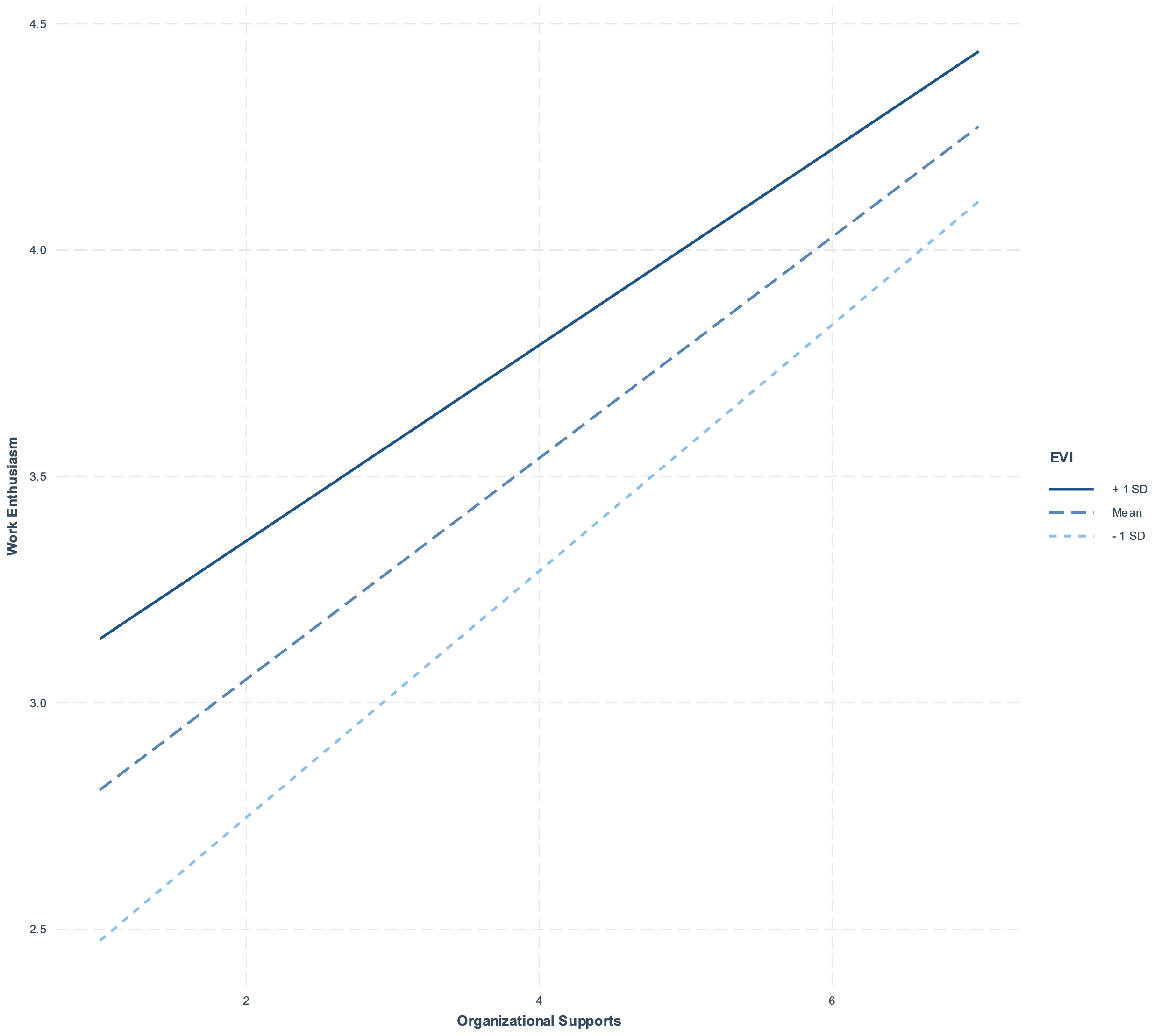


### Turnover Intention

Figure S3.5. Plot of the interaction of Organizational Supports and EVI predicting Turnover Intention.

###
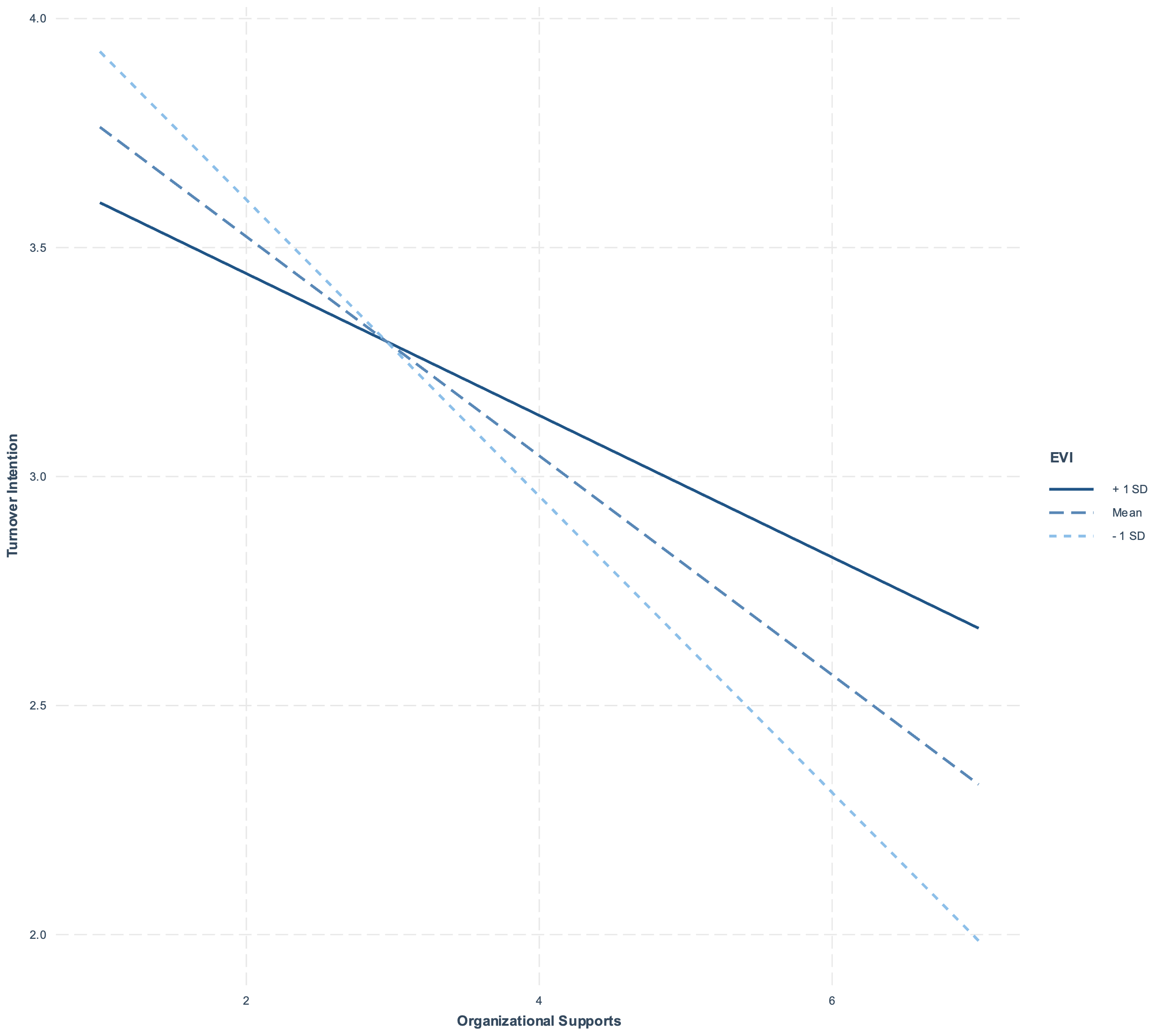


Figure S3.6. Plot of the interaction of Workplace Stress and EVI predicting Turnover Intention.


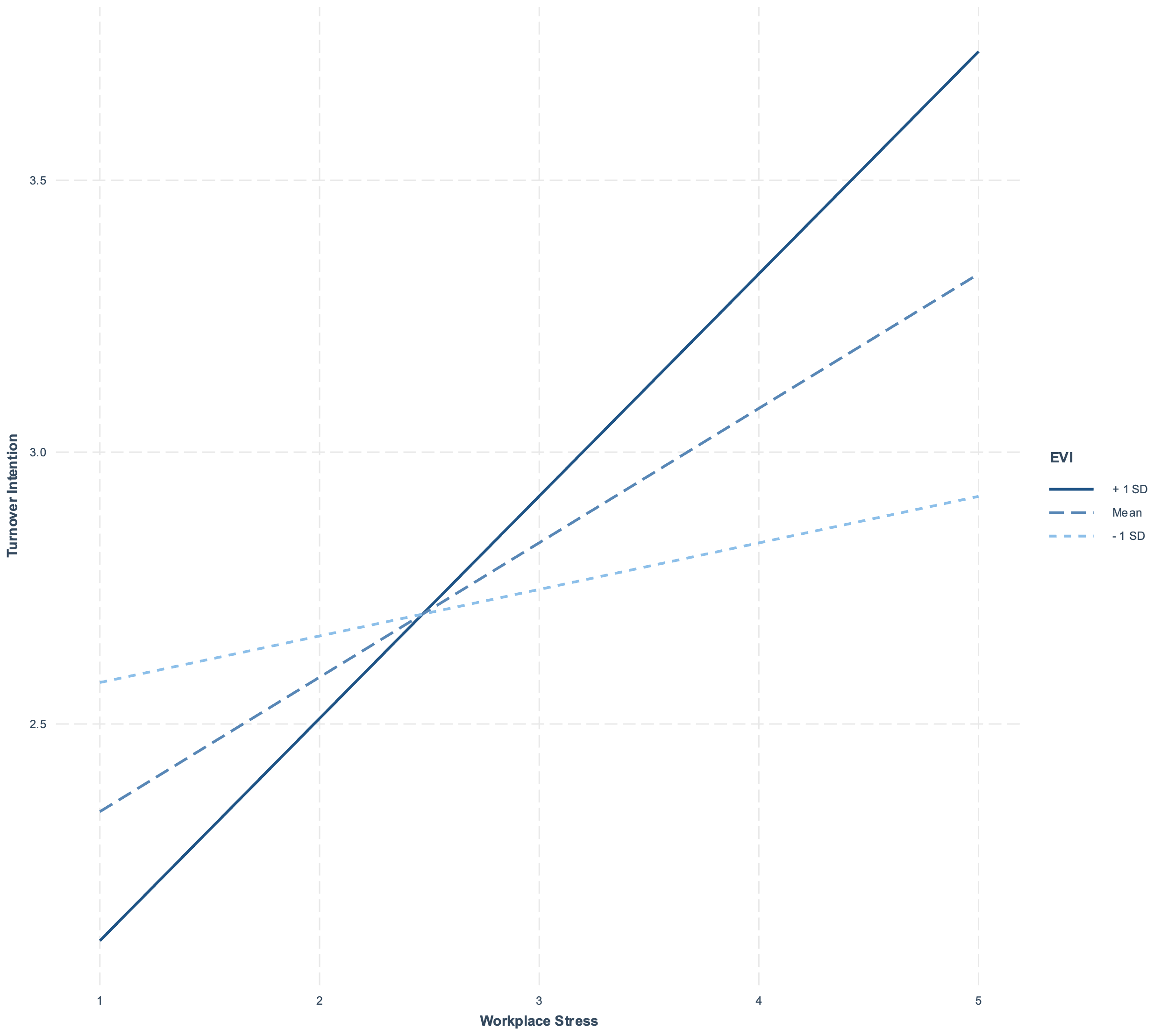


## References

Hayton, J. C., Allen, D. G., & Scarpello, V. (2004). Factor retention decisions in exploratory

factor analysis: A tutorial on parallel analysis. *Organizational Research Methods, 7*(2), 191-205. https://doi.org/10.1177/1094428104263675
